# Sleep/Wake Dynamics Changes during Maturation in Rats

**DOI:** 10.1371/journal.pone.0125509

**Published:** 2015-04-20

**Authors:** Gideon Gradwohl, Nilly Berdugo-Boura, Yael Segev, Ariel Tarasiuk

**Affiliations:** 1 Sleep-Wake Disorders Unit, Soroka University Medical Center and Department of Physiology, Faculty of Health Sciences, Ben-Gurion University of the Negev, Beer-Sheva, Israel; 2 Shraga Segal Department of Microbiology and Immunology, Faculty of Health Sciences, Ben-Gurion University of the Negev, Beer-Sheva, Israel; 3 Unit of Biomedical Engineering, Department of Physics, Jerusalem College of Technology, Jerusalem, Jerusalem, Israel; University of California, Riverside, UNITED STATES

## Abstract

**Objectives:**

Conventional scoring of sleep provides little information about the process of transitioning between vigilance states. We applied the state space technique (SST) using frequency band ratios to follow normal maturation of different sleep/wake states, velocities of movements, and transitions between states of juvenile (postnatal day 34, P34) and young adult rats (P71).

**Design:**

24-h sleep recordings of eight P34 and nine P71 were analyzed using conventional scoring criteria and SST one week following implantation of telemetric transmitter. SST is a non-categorical approach that allows novel quantitative and unbiased examination of vigilance-states dynamics and state transitions. In this approach, behavioral changes are described in a 2-dimensional state space that is derived from spectral characteristics of the electroencephalography.

**Measurements and Results:**

With maturation sleep intensity declines, the duration of deep slow wave sleep (DSWS) and light slow wave sleep (LSWS) decreases and increases, respectively. Vigilance state determination, as a function of frequency, is not constant; there is a substantial shift to higher ratio 1 in all vigilance states except DSWS. Deep slow wave sleep decreases in adult relative to juvenile animals at all frequencies. P71 animals have 400% more trajectories from Wake to LSWS (*p* = 0.005) and vice versa (*p* = 0.005), and 100% more micro-arousals (*p* = 0.021), while trajectories from LSWS to DSWS (*p* = 0.047) and vice versa (*p* = 0.033) were reduced by 60%. In both juvenile and adult animals, no significant changes were found in sleep velocity at all regions of the 2-dimensional state space plot; suggesting that maturation has a partial effect on sleep stability.

**Conclusions:**

Here, we present novel and original evidence that SST enables visualization of vigilance-state intensity, transitions, and velocities that were not evident by traditional scoring methods. These observations provide new perspectives in sleep state dynamics and highlight the usefulness of this technique in exploring the development of sleep-wake activity.

## Introduction

It has long been recognized that adequate sleep is fundamentally important for healthy growth, development, and homeostasis in human animals [1–5]. During maturation there is a reduction of total sleep duration by about 10–20% between ages 10 and 20 years [6–9] and decreased delta sleep power [10–12]. Studies using traditional categorical sleep state scoring revealed that maturation can lead to increased wake time, decreased paradoxical sleep (PS), and decline of slow wave sleep (SWS) duration and power [13,14]. Several studies in rodents have reported an increase in wakefulness during puberty, which was complemented by a decrease in SWS and PS duration [15] and slow wave activity [14,16,17]. The amplitude and slope of electroencephalography (EEG) slow waves is related to the number of neurons that enter an up state or a down state near-synchronously, and that synchrony is directly related to the number and efficacy of synaptic connections among them [18–21]. Research on age-related sleep in several species demonstrated that there are age-associated changes in the synaptic distribution and connection strength [22–25]. Recently it was demonstrated that in the mouse cortex the observed developmental decrease in sleep slow wave activity cannot be accounted for simply by a net pruning of synapses [16].

Traditional sleep scoring reveals little information about the process of transitioning between vigilance-states in humans and animals [4,5,21,26,27], as cortical activity and behavior can change quite rapidly in rodents. The traditional scoring methods simply identify discrete states at a time window (4 to 10 seconds), so they can overlook important variations within states, or often can exclude or dilute events through averaging; and therefore rapid changes between these states or within a specific state may be disregarded. The 2-dimensional (2D) state space technique (SST) provides a novel unbiased approach, a non-categorical method for comparison and quantitative analysis of sleep/wake states [28–30]. The previous application of SST of sleep recordings used local field potential data, but the variability in these signals prevented comparisons between animals [31–33]. Recently the SST was further developed to enable inter-animal comparisons of EEG dynamics of sleep/wake behavior [28–30]. SST has high temporal resolution and analyzes behavior as a continuum, rather than discrete states, thus facilitating higher dimensional examination of state transitions that are not possible with traditional methods. Traditional spectral analysis often excluded or diluted events through averaging. In addition, this approach employs the ratio of two frequency bands rather than a single frequency band.

We hypothesize that SST can highlight the richness of sleep states transitions and the states’ stabilities as a continuum physiological process rather than the traditional and categorical scoring of sleep/wake behavior, and so get new novel insights into sleep physiology. The effect of maturation in rats on electroencephalography (EEG) dynamics of vigilance-states using the SST was not explored. The current study analyzes, by using SST, the impact of maturity on sleep/wake dynamics, sleep stage stability, and movements between states.

## Methods

### Animals

Two groups of animals were used: young (postnatal day 34, P34) and adult (P71) male rats (Sprague-Dawley, Harlan Laboratories Inc., Jerusalem). Animals were kept on 12–12 light-dark cycle with lights on 09:00, 23±1.0°C [4,5]. Rats were acclimatized to the recording boxes for 7 days prior to recordings. Food and water were given *ad libitum*. The study was approved by the Ben-Gurion University of the Negev Animal Use and Care Committee and complied with the American Physiological Society Guidelines.

### Surgery

Animals were anesthetized with tribromoethanol (200 mg/kg) administered intraperitoneally. A telemetric transmitter (TL11M2-F20-EET Data Sciences International, DSI, St. Paul, MN, USA) was implanted under sterile conditions, enabling recording of EEG, dorsal neck electromyography (EMG), and body temperature. Leads from the electrodes for EEG recording were placed over the frontal (1.1 mm anterior and 1.1 mm lateral to the bregma) and parietal (3 mm posterior and 1.5 mm lateral to the bregma) cortices. EEG electrodes were anchored to the skull with dental cement [4,5]. Following surgery, prophylactic enrofloxacin 5 mg/mL (s.c.) and water containing ibuprofen (0.1 mg/mL) were given for three days [4,5,34].

### Sleep-wake recording

Sleep was recorded 7 days following recovery from surgery when the animals had acclimatized to the recording environment. Raw EEG and EMG outputs from the skull and skeletal muscle electrodes were sampled at 250 Hz, filtered at 0.5–40 Hz and 10–250 Hz, respectively, using DSI system [4,5,28].

### Conventional scoring of vigilance-states

Vigilance-states were scored using NeuroScore software (v. 2.1 software, DSI St. Paul, MN, USA) and edited visually for 10-second epochs on the basis of the predominant state within the epoch [4,5,35,36]. The duration of sleep-wake states was calculated for 12-hours light, dark, and for the whole 24 hours. The states of vigilance were categorized as: 1) Wake, 2) SWS, and 3) PS. Only epochs of quiet wakefulness when animals are not active were scored. SWS—high-amplitude EEG waves, lack of body movement, and predominant EEG power in the delta range (0.5–4.0 Hz); Light SWS (LSWS)—high-voltage slow cortical waves (0.5–4 Hz) interrupted by low-voltage fast EEG activity (spindles, 6–15 Hz). Deep SWS (DSWS)—continuous (>70% epoch) high-amplitude slow cortical waves (0.5–4 Hz) with reduced EMG and motor activity. PS—highly regular low-amplitude EEG, dominance of theta activity with corresponding high fast theta (5.0–8.0 Hz) power, general lack of body movements with occasional twitches; Wake—less regular low-amplitude EEG, lack of visible theta dominance, and frequent body movements. The amplitude and power density of the EEG were calculated during 24 hours for 10-sec epochs in the frequency range 0.5–40 Hz (Hanning window) and frequency resolution 0.5 Hz [5]; data were averaged separately for each vigilance state.

### Construction of the 2D state space

We defined a 2D state space using 2 spectral amplitude ratios calculated by dividing integrated spectral amplitudes at selected frequency bands [29,30]. A sliding window Fourier transform was applied to each raw (0.5–40 Hz) EEG signal using a 2-second window with a 1 second step size. Then we calculated two spectral amplitude ratios by integrating the spectral amplitude over specific frequencies: 0.5–20/0.5–40 Hz for ratio 1 (plotted on the abscissa) and 0.5–4/0.5–9 Hz for ratio 2 (plotted on the ordinate). We used these ratios according to previously reported methods [31]. These heuristic ratios, determined after a careful search of parameters for the numerator and denominator, enable the best separation of vigilance states. Since the frequency range of the numerator was included in the denominator, the ratios are between 0 and 1. The ratios derived from 24-hour EEG recordings were smoothed with a 20-second wide Hanning window. Each second of this smoothed ratio was mapped into the 2D state space, ratio 2 vs. ratio 1. A graphical user interface was developed to validate the 2D SST by comparing the clustering of 2D state space against the conventionally scored vigilance-states. A general agreement between these two methods was found; distinct clusters of points correspond to distinct states of Wake, SWS, and PS. SST yields Wake at low ratio 1 values and SWS at high ratio 1 values.

### State space densities

To calculate state space densities, the state space was binned using a grid of 1000 × 1000 uniformly spaced boxes for each animal separately. Then the individual grids were summed and averaged, to produce the density graph of the “average” animal. This state space densities graph was projected to ratio 1. The densities plotted due to this projection reflect the distribution of the sum of ratio 2 vs. ratio 1. Furthermore, in order to estimate the duration of each vigilance state, the area under the projection plot (integral) was calculated.

### State space velocities

For each animal, we created state space velocity maps to determine rates of change across the state space. The speed of the spectral change was calculated as the distance between two consecutive data points (calculated from the horizontal and vertical velocities by the Pythagorean Theorem), and in this sense, the temporal resolution was second by second. After this calculation, we mapped the velocities to a 1000 × 1000 grid with the values at each point representing the average velocities originating at that site [28].

### Trajectory analysis

To understand the patterns of EEG activity as rats move between stable states, we identified trajectories in the state space by tracking consecutive sequences of points. First, for each vigilance-state the state space densities were plotted and all values up to 20% of the maximum value were included in a boundary for each vigilance-state. Trajectories connecting different clusters were considered to be valid transitions if: 1) the duration of the trajectory connecting two different clusters was 60 seconds and 2) the trajectory spent at least half of the preceding 30 seconds in the initiating cluster and half of the 30 subsequent seconds in the terminating cluster. In addition, we explored the micro-arousal change due to maturation during 24 hours [28,31].

### Data analysis

Sleep/wake dynamics and statistical analyses were performed using MATLAB (R-2008b, The MathWorks, Inc., Natick, MA, USA). Significance was analyzed by Student’s *t*-test or Wilcoxon Rank-sum test as appropriate. Two-way analysis of variance for repeated measures was used to determine significance between frequency and group, for *post hoc* comparisons by Student–Newman–Kuels test. Null hypotheses were rejected at the 5% level.

## Results

### Vigilance-states

Traditional scoring of vigilance-states indicated that both groups exhibited circadian rhythms of Wake, SWS, and PS. Adult animals have about 50% more LSWS during the light on (*p* = 0.006) and 24 hour (*p* = 0.02) periods (Table 1). This was complemented by 68% reduction of DSWS (*p* = 0.03) in the light period. Adult animals have 43% and 30% less PS in the dark and 24-hour periods, respectively (*p* < 0.05). Wake amplitude and power density of adult animals decreased by 19.2% and 31.6% at 8 Hz compared with juvenile rats, respectively (*p* < 0.001), see S1A and S1B Fig. LSWS amplitude and power density of adult animals decreased by 14.9% and 31.5% at 2.5 Hz compared with juvenile rats, respectively (*p* < 0.001). DSWS amplitude and power density of adult animals decreased by 28.3% and 50.0% at 2.5 Hz compared with juvenile rats, respectively (*p* < 0.001). PS amplitude and power density decreased by 19.2% and 29.4% at 7.5 Hz compared with juvenile rats, respectively (*p* < 0.001).

**Table 1:** Spontaneous sleep in juvenile and adult rats.

	Light period		Dark period		24-Hour period	
	P34	P71	P34	P71	P34	P71
Wake (%)	33.5±6.5	43.8±4.6	48.7±9.2	53.0±6.3	41.9±7.6	50.4±4.2
SWS (%)	52.4±6.7	44.4±3.2	38.1±8.5	35.1±5.9	46.4±7.5	40.7±3.4
PS (%)	13.3±1.9	9.8±1.6	8.1±0.8	4.6±1.3*	11.0±0.8	7.6±1.2*
LSWS (%)	23.9±2.5	35.5±2.5#	9.1±1.6	13.0±4.5	17.0±1.8	25.8±3.0*
DSWS (%)	28.5±6.7	8.9±5.0*	29.0±7.7	24.6±8.3	29.4±3.1	14.9±5.0

Average % of time spent in each sleep stage for light phase (09:00–21:00) and dark phase (21:00–09:00). SWS—slow wave sleep; PS—paradoxical sleep; SWA—slow wave activity; DSWS—deep slow wave sleep; LSWS—light slow wave sleep; values are mean ± SEM.

*p* <0.05

# *p* < 0.006 comparing juvenile with adult animals.

### State space technique (SST)

After applying the SST, the distinct vigilance states (Wake, SWS, and PS) are organized as clusters, corresponding to the traditional scoring of these states, and located at different locations in the 2D state space (S2A–S2D Fig). Wake clusters occurred at small ratio 1 values, while SWS clusters occurred at high ratio 1 values. PS appeared between Wake and SWS clusters. The distribution of points within each cluster reflected variations in sleep depth and intensity or the strength of wakefulness.

**Fig 1:**
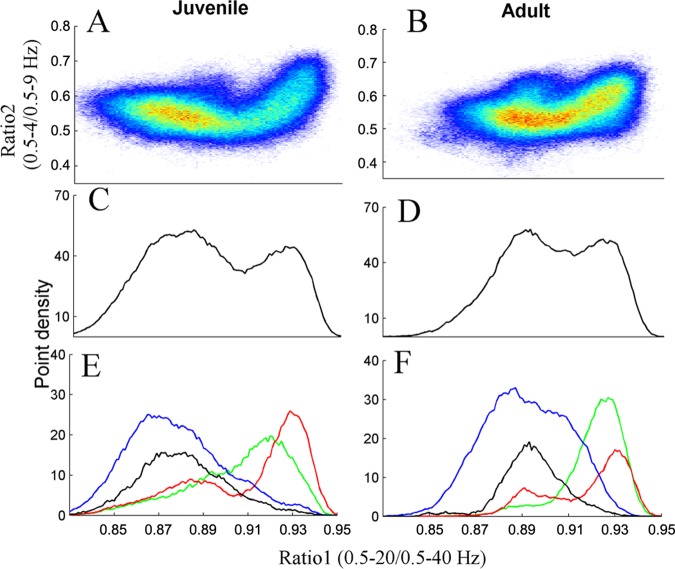
Point densities averaged across all control (A) and UAO (B) animals of the 2D state space plots. Each plot showed 24 hours of EEG activity, and each point represented 1 second of EEG activity, after application of 20-second wide Hanning window. (C), (D)–“average” state space densities for juvenile and adult animals, projected into ratio 1, respectively. This projection yielded two peaks; left peak was associated with Wake and PS, and the right peak was associated with SWS sleep. (E), (F) Each of the vigilance state space point densities (not shown) was projected separately into ratio 1. Blue—Wake; Black—paradoxical sleep (PS); Green—light slow wave sleep (LSWS); Red—deep SWS (DSWS).

Point densities of the 2D state space plots of an “average” animal (see Methods) are presented in Fig 1. Fig 1A and 1B presents the results of juvenile and adult animals, respectively. Warm colors indicate regions where the average density is high and cool colors specify low density. To analyze the point densities they were projected into ratio 1 (Fig 1C and 1D) and this projection revealed two peaks. To further emphasize the contribution of each of the vigilance states to the overall projection into ratio 1, the density graph for each vigilance-state of the "average" animal was produced (not shown) and projected separately (Fig 1E and 1F). Comparing the vigilance-states projections (Fig 1E and 1F) to the overall projection (Fig 1C and 1D) indicates that the left peak (low ratio 1) is composed mainly of wake and PS, while the right peak is SWS states (high ratio 1).

**Fig 2:**
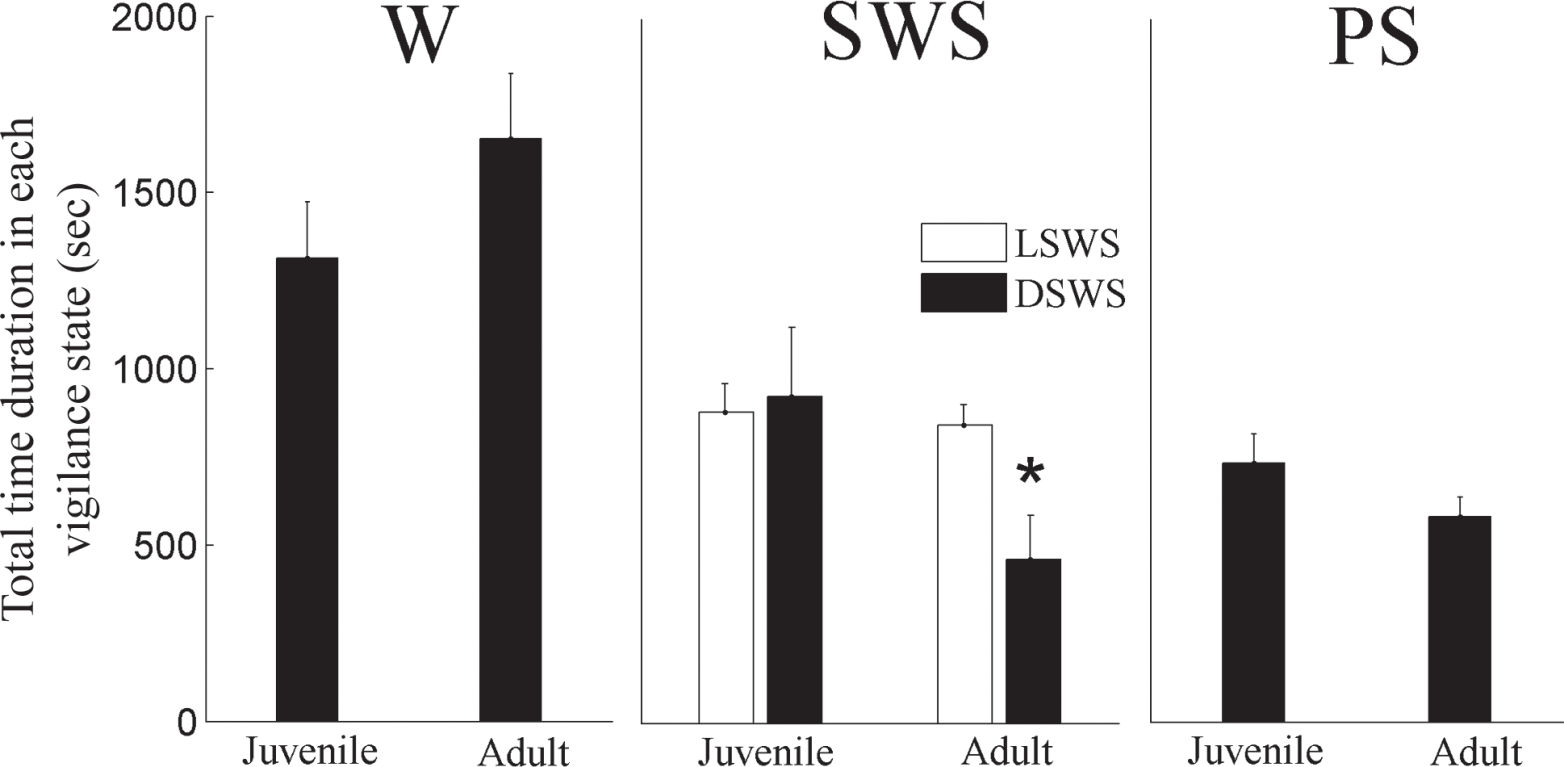
Total time duration in each vigilance state. Time was calculated by integrating the area under the curve of each vigilance state illustrated in Fig 1E and 1F. LSWS—light slow wave sleep; DSWS—deep SWS. W—Wake, PS—paradoxical sleep. * *p* < 0.021—comparing LSWS to DSWS in adult rats.

To further analyze our results we calculated density graphs and their projection for each animal. Analyzing the horizontal distance between peaks of the density graph of all vigilance states yielded no significant result; it was 27.8±25.0 (arbitrary units) and 22.6±14.8 (arbitrary units) in juvenile and adult rats, respectively (*p* = 0.626). Therefore each vigilance state was analyzed separately. The peak amplitude of LSWS of juvenile and adult rats was 30.2±6.7 (arbitrary units) and 42.4±4.5 (arbitrary units), respectively (*p* = 0.001), with no significant change in ratio 1 location. DSWS peak location and its amplitude were similar in both groups. PS peak location on ratio 1 of juvenile and adult rats was 875.9±12.2 (arbitrary units) and 895.7±10.1 (arbitrary units), respectively (*p* = 0.005). Wake location on ratio 1 of juvenile and adult rats was 870.0±12.6 and 885.1±13.8, respectively (*p* = 0.005). The peak amplitude of Wake of juvenile and adult animals was 40.1±7.5 (arbitrary units) and 52.9±3.6 (arbitrary units), respectively (*p* = 0.001). Thus, the cause of the horizontal distance (between the peaks in Fig 1C and 1D) reduction due to maturation is the shift of the Wake and PS peaks (with no movement of SWS peaks) to higher ratio 1 values. Fig 2 summarizes the time duration (integral of the projection curve) for each vigilance state. DSWS decreases due to maturation by 45% compared to LSWS (*p* = 0.021).

**Fig 3:**
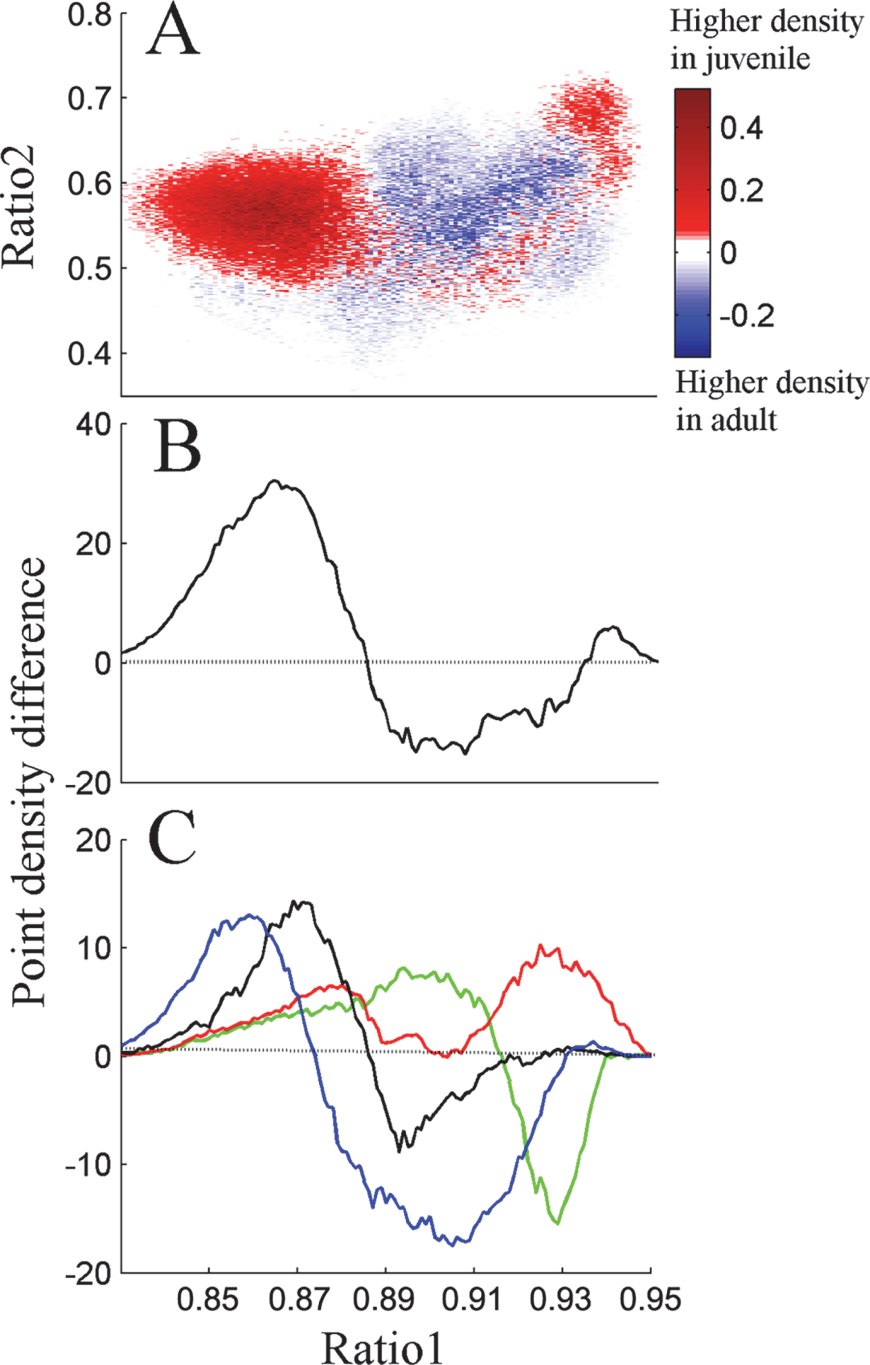
Difference plot showing average density pattern of the “average” adult rat subtracted from that of the “average” juvenile rat (A). The color scale highlights differences between the groups: warm colors indicate regions where the average density is higher in juveniles and cool colors indicate higher density in adult group. Projection of this graph into ratio 1 is presented in (B). (C) Each of the vigilance states of the “average” difference plot (not shown) were projected separately into ratio 1. Positive values in (B) and (C) indicate higher density values for juvenile and negative higher density in adult. Blue—Wake; Black—paradoxical sleep (PS); Green—light slow wave sleep (LSWS); Red—deep SWS (DSWS).

To further understand the effect of maturation on vigilance-states, the average density plot of the "average" adult rat was subtracted from the "average" juvenile rat density plot (Fig 3A); we plotted the projection of this graph to ratio 1 (Fig 3B). The juvenile rats had prominent increased cluster densities at smallest ratio 1 values (indicated by the red region in the left side of Fig 3A, or by the positive values in Fig 3B), whilst adult rats relative to juvenile rats had higher density clusters at larger ratio 1 values (blue region at the center in Fig 3A or by the negative values in Fig 2B). At the largest ratio 1 value the juvenile rats had slightly higher density clusters than the adult rats (red region at the right side of Fig 3A). To further quantify the positive and negative curves and to explore their components, we analyzed the projections of the vigilance-states (Fig 3C) based on the difference graph of each vigilance-state density graph (not shown). It appears that in adults all density clusters (except DSWS) shifted to higher ratio 1 value relative to juvenile rats. However in adults, DSWS cluster density decreased at all ratio 1 values. To ensure that the inclusion of PS did not affect our findings, we eliminated data scored as PS sleep and reanalyzed the data. The results of that analysis were very similar (data not shown). In order to understand the shift to higher ratio 1 values in adult rats, the 24-hr mean EEG amplitude integrals (for each vigilance state separately) based on S1 Fig, were calculated between 0.5 and 20 Hz and 0.5 and 40 Hz for the numerator and denominator, respectively. In adult animals both the numerator and denominator decrease by 20% to 30% in comparison to juvenile animals in all vigilance states except for DSWS (*p* < 0.05). However, the denominator decreased more than the numerator by about 1% to 2% (*p* < 0.05). Despite the decrease of the numerator and denominator in adult animals, their division yielded higher ratio 1 values than in juvenile animals.

### State space velocities

**Fig 4:**
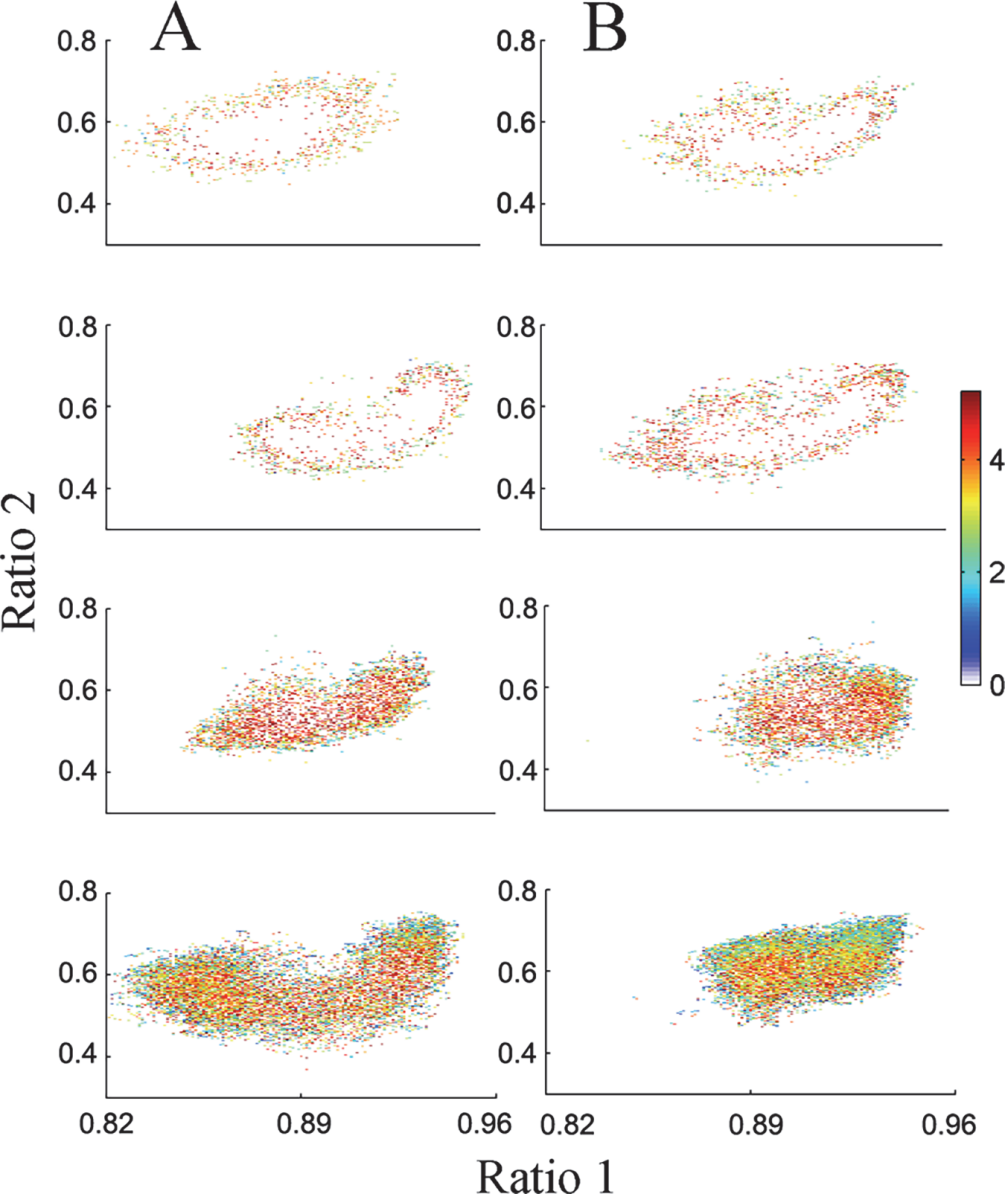
Maturation did not alter velocity. Velocity is defined as the horizontal and vertical distance between two consecutive points in the point density plot divided by the temporal resolution of 1 second. Plots represent velocities calculated by Pythagorean Theorem for four juvenile (A) and adult (B) animals. Values represent average velocities originating at that site. Warm colors show fast velocities (transition regions) and cool colors slow velocities.

**Fig 5:**
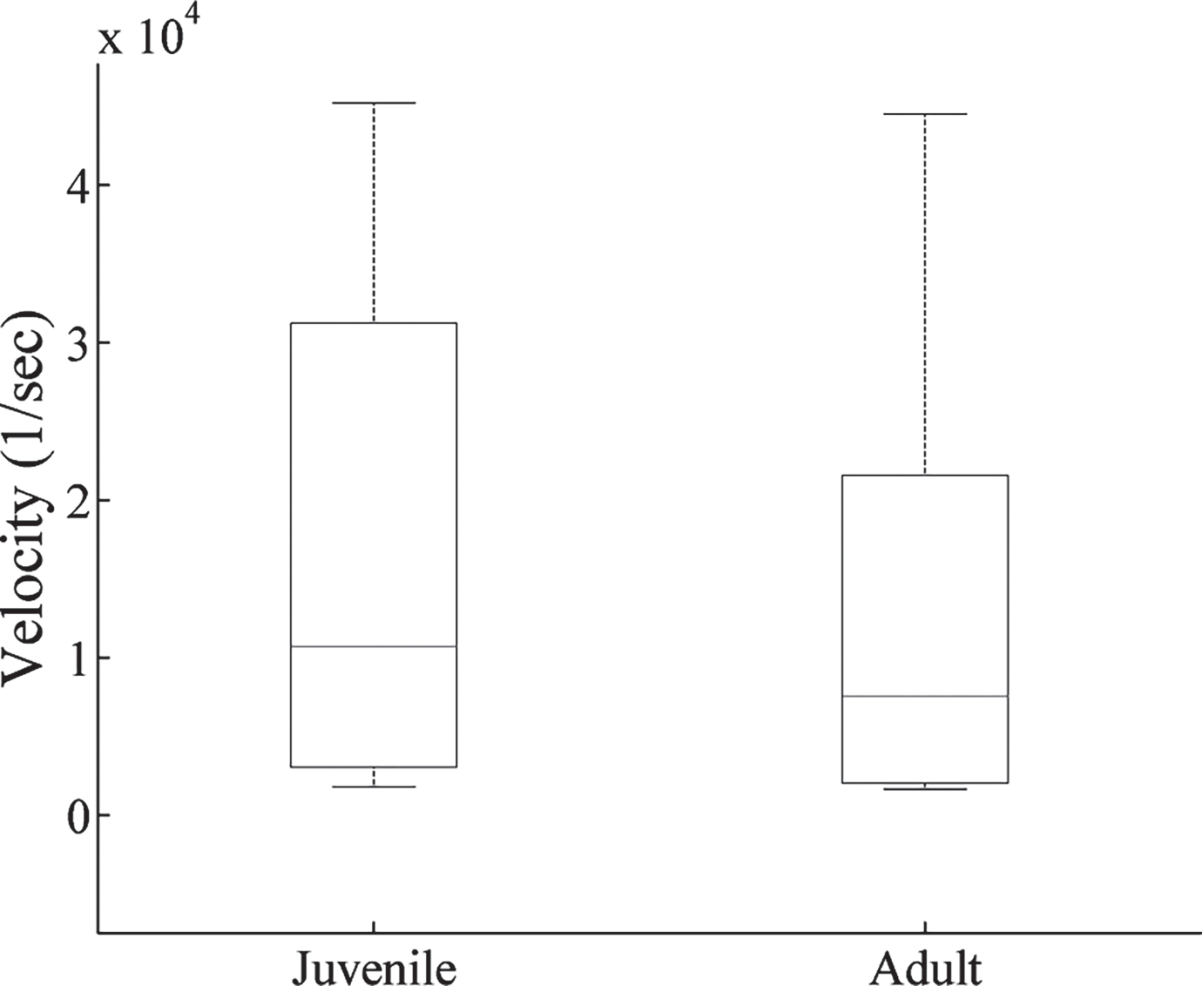
Median velocity of juvenile and adult animals. Box plot shows the median velocity following calculation of the sum of the velocities in each animal.

Fig 4 illustrates an example of individual velocity plots for 4 juvenile (Fig 4A) and 4 adult (Fig 4B) animals. The velocities of both groups were similar at all regions of the 2D state space plot with no significant differences in the median velocity (Fig 5).

### Trajectories between vigilance-states

To explore the patterns of 2D state space dynamics as rats move between vigilance-states, we identified trajectories in the 2D state space by tracking consecutive sequences of points. Using a contour algorithm (see Methods), we delineated cluster core boundaries for each possible vigilance-state (Table 2). For all vigilance-states, all moving combinations of trajectories were calculated. In adult rats the total number of trajectories increased by 58% (*p* = 0.022). In adult rats the most common trajectories were between Wake and LSWS and vice versa, 30.0% and 23.2%, respectively, while in juvenile rats the trajectories between LSWS and DSWS and vice versa were 40.9% and 27.6%, respectively.

**Table 2:** Mean number of trajectories between vigilance-states.

		Juvenile (P 34)	Adult (P 71)	*p* value
		(Mean, %)	(mean, %)	
Total number of trajectories		150.1±26.3 (100%)	237.3±21.3 (100%)	0.022
From stage	To stage			
Wake	LSWS	16.9±4.2 (11.2%)	71.2±15.1 (30.0%)	0.005
LSWS	Wake	13.4±3.4 (8.9%)	55.1±12.1 (23.2%)	0.007
LSWS	DSWS	40.9±10.5 (27.2%)	15.7±5.8 (6.6%)	0.047
DSWS	LSWS	27.6±6.7 (18.4%)	10.3±3.6 (4.4%)	0.033
LSWS	PS	7.4±1.7 (4.9%)	13.2±3.6 (5.6%)	0.178
Wake	DSWS	5.4±2.2 (3.6%)	13.2±9.3 (5.6%)	0.448
DSWS	Wake	4.9±2.2 (3.2%)	8.4±5.1 (3.6%)	0.55
Wake	PS	5.0±3.0 (3.3%)	7.9±1.6 (3.3%)	0.392
PS	LSWS	8.1±2.6 (5.4%)	5.7±2.8 (2.4%)	0.533
DSWS	PS	2.9±1.0 (1.9%)	2.8±1.5 (1.2%)	0.958
PS	Wake	8.8±3.0 (5.8%)	2.0±0.6 (0.8%)	0.035
PS	DSWS	0.9±0.4 (0.6%)	1.2±0.7 (0.5%)	0.696

Data show the number of spontaneous transitions between behavioral states collected from 456 hours of a rat’s life. W—Wake; LSWS—light slow wave sleep; DSWS—deep slow wave sleep; PS—paradoxical sleep. Values are mean ± SEM. (%) Percent of total number of trajectories.

Comparing the effect of maturation on trajectories from Wake to LSWS and vice versa in adults, yielded results that were significantly 412% and 411% higher, respectively (Table 2). In comparison, the number of trajectories from LSWS to DSWS and vice versa in adults relative to the juvenile group was 63% and 44% smaller, respectively. Although the trajectories between PS and Wake were rarely used, adults had significantly 78% fewer trajectories than in the juvenile rats.

**Fig 6:**
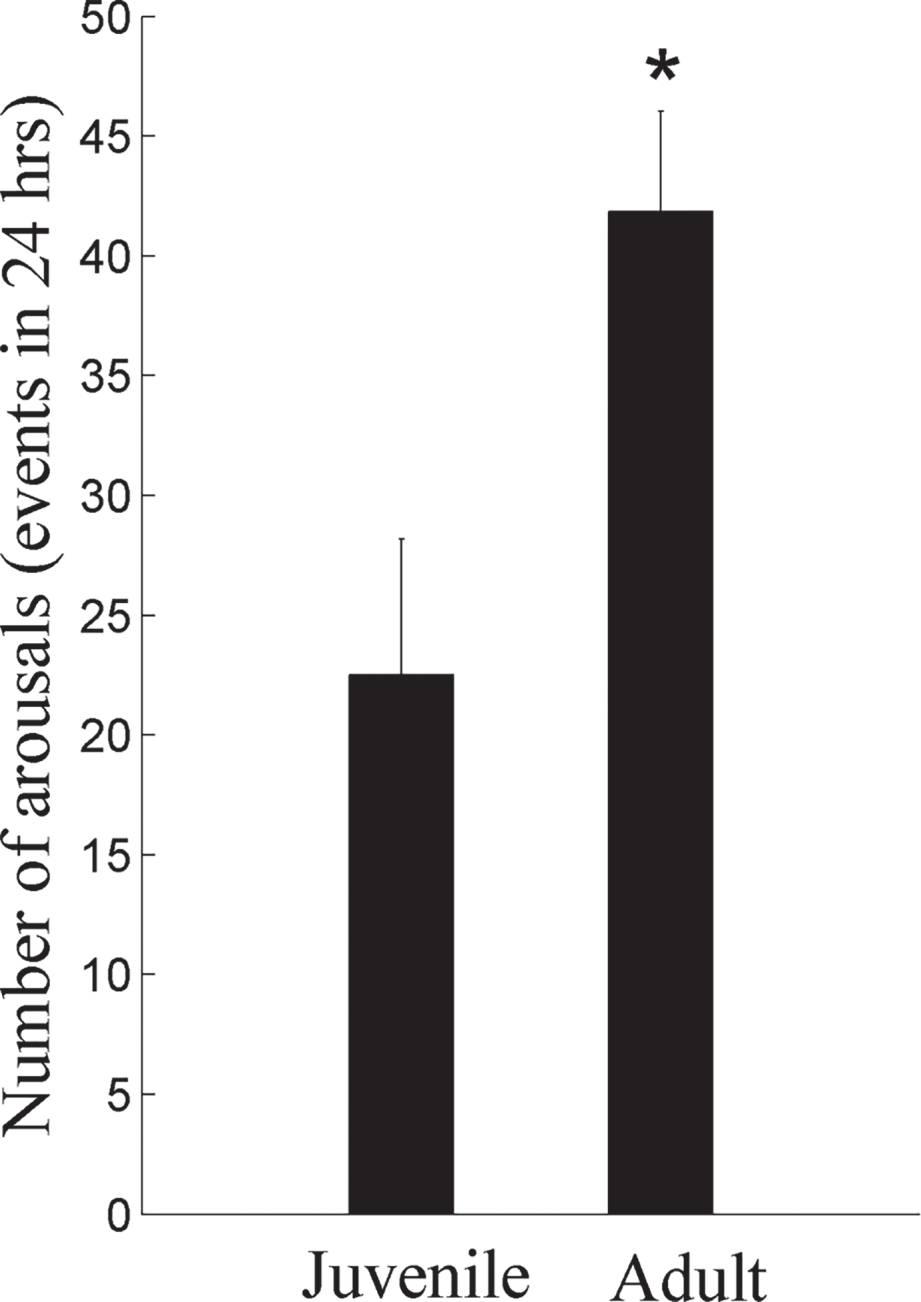
Effect of maturation on micro-arousals during 24 hours. Adult animals had significantly more micro-arousals (≥3 up to 15 seconds) in the whole day. * *p* < 0.021.

In both groups, only a negligible number of micro-arousals (from sleep to Wake) had duration of less than 3 seconds, and therefore we defined a micro-arousal event as one that lasted in the range of ≥3 to 15 seconds [29]. In the adults the number of micro-arousals increased by 100% relative to juvenile rats (Fig 6; *p* = 0.021). Most of the micro-arousal events in juvenile and adult rats, 70% and 54%, respectively, occurred during LSWS.

## Discussion

To our knowledge this is the first study exploring the effects of maturation on sleep dynamics using SST. We present evidence that with maturation vigilance state determination, as a function of frequency range, is not constant; there is a substantial shift to higher ratio 1 values in all vigilance states except DSWS. Both EEG power and amplitude decreased at all frequency ranges with maturation at all vigilance states (S1C and S1D Fig). With maturation there are considerably more trajectories from Wake to LSWS and vice versa, and an increased number of micro-arousals, while trajectories from LSWS to DSWS and vice versa were considerably reduced. In both juvenile and adult animals, no significant changes were found in velocity at all regions of the 2D state space plot. These findings suggest that maturation has a partial effect on sleep stability, alteration of micro-arousals, and no effect on velocity.

Since independent life in the laboratory begins at artificial weaning, some researchers use weaning to define the beginning of adolescence, allowing overlap with life events associated with young childhood in humans [16,37,38]. In our study we used rats at age P34 early adolescence and P71 young adult comparable to about 8 [4] and >20 [5] years in humans. During maturation, the brain undergoes critical developmental and hormonal processes [15,36,37,39], which are characterized by extensive morphological and functional changes. Traditional sleep scoring revealed that SWS duration, power, and slow wave activity decrease across puberty [13–18]. The decline of SWS may reflect developmental changes of the brain [10,11,14–17,37]. Similar to earlier reports, conventional sleep scoring in our study revealed that SWS duration did not change with maturation (Table 1). However, the duration of LSWS increases and DSWS decreases during lights-on (Table 1) and during 24 hours (Fig 2). We used SST to capture the richness of SWS better than the conventional methods. SST analysis of sleep intensity showed that at high ratio 1 value adult animals have notable increase of LSWS (negative green curve in Fig 3C), while at the same time DSWS decreases as indicated by the positive red curve in Fig 3C. As indicated by using our graphical user interface, SWS region is on the right side of the 2D SST graph, while wake cluster is on the left side (S2A and S2B Fig). With maturation there is a shift of LSWS to higher ratio 1 values as noted by the positive green peak (ratio 1 value ~0.9) and negative green peak (ratio 1 value ~0.93). Since wake frequency range is higher than SWS, a shift to high ratio 1 (towards the SWS region) suggests reduction of frequency.

In our study, a novel approach using SST enables the analysis of sleep stage stability and EEG dynamics. Trajectories connecting different clusters in the 2D-state space densities of each vigilance-state value were calculated using one-second resolution. Maturation leads to considerably more trajectories between vigilance states during the 24 hours (Table 2), supporting recent reports on mice [40]. In adult animals most trajectories moved from Wake to LSWS and vice versa, while trajectories from LSWS to DSWS were less common and more frequent micro-arousals were observed. This suggests that sleep maturity leads to more shallow sleep. In contrast, during chronic upper airway obstruction in rats [28] and orexin knockout mice [30], sleep has more regions with unstable vigilance-states, as indicated by increased velocity at the 2D state space plot. In our study a large variability in the SST velocity was observed in both ages (Figs 4 and 5); this finding is in accordance with previous reports [28, 30]; further studies are required to explore this finding.

In the current study artifact-free epochs of quiet Wake were analyzed. By using the SST, it was possible to capture the richness of temporal changes in vigilance states [28–31], which to our knowledge were never reported before. Considerable reduction of Wake intensity was found (high ratio 1 values) with maturation (Fig 3), while Wake duration did not change significantly (Table 1). This finding is illustrated by the left red cluster in Fig 3A and the blue positive curve in Fig 3C, and the following blue cluster in Fig 3A and negative blue curve in Fig 3C. This observation was not affected by the presence of PS, since elimination of PS from the analysis revealed similar results (data not shown). Several possibilities may explain our finding:

It was hypothesized that Wake is associated with a net increase of synaptic strength that decreases during sleep [41]. Enhancement of synaptic strength may be due to greater density and/or efficacy of cortical synapse. During early childhood, neurons grow and become more branched dendritic trees with an increased number of synapses. Then, during adolescence the net number of synapses decreases and the synaptic number of connections to other neurons diminishes. However, a two-photon microscopy study reported no change in spine loss or gain across Sleep and Wake changes as a function of age during early to late adolescence [42]. Orexin neurons are typically active during wakefulness (lights off) but show little or no activity during sleep [43–45]. Several studies demonstrated reduction of orexin neuron density and receptor 2 mRNA during maturation in humans [46] and mice [47]. These observations support our finding that Wake intensity declines in adult animals.

### Study limitation

SST is especially useful for studying the dynamics of sleep/wake instability, but it has some limitations as previously discussed in several studies [28–31]. Using frequency ratios of parameters could yield hypersensitivity; small changes of the denominator over time may lead to huge fluctuations in the corresponding ratio. Converting the ratios to the log of a ratio will produce small deviations of either the denominator or the numerator, producing small changes in the 2D state density plot [29]. Another limitation can be that at different hours the density graphs yield distinct boundaries. Therefore it should be recognized that comparison of trajectories and micro-arousals between different hours could be problematic since values are calculated according to these boundaries. Furthermore, the division method could be problematic; SST relies on the 10-second epoch of vigilance state determination and was divided to ten parts of 1 second each for greater temporal resolution within the same state. However, according to this partition we neglect the potential short fluctuations of the sleep behavior. Also, the separation between the Wake and PS clusters was less well-defined using the surface EEG signal [31]. Previous work has shown a high degree of coherent activity in cortex, hippocampus, striatum, and thalamus across states in normal animals [31] and humans [48], suggesting that a single channel of EEG is sufficient for state space analysis of sleep/wake behavior. Future investigations with larger age range and a more detailed EEG montage could be used to determine the effects of maturation on cortical coherence and its contributions to sleep/wake dynamics.

## Supporting Information

S1 Fig Electroencephalogram amplitude (A) and its power density (B). Values were calculated in wake (W), light slow wave sleep (LSWS), deep slow wave sleep (DSWS), and paradoxical sleep (PS) during 24 hrs in juvenile (open symbols) and adult (closed symbols) rats. Each point represents mean values of 0.5 Hz bins in a 24-hr recording period. There are statistically significant differences between the groups in both amplitude and power density at all frequencies in all vigilances states, *p* < 0.0001 (two-way ANOVA). Values are mean ± SEM.
(TIF)

S2 Fig Spectral ratios of EEG activity define a 2-dimensional state space with distinct clusters. (A) Juvenile and (B) adult rat; spectral ratios of EEG activity define a 2D state space with distinct clusters. Each plot shows 24 hours of EEG activity, and each point represents 1 second of EEG activity. (C) and (D) show 2D state space after application of a Hanning window (20 seconds) of one juvenile and one adult animal, respectively. Blue—wake; Black—paradoxical sleep (PS). Green—light slow wave sleep (LSWS); Red—deep SWS (DSWS).
(TIF)
