# Supplementary figures and images for: Sleep/Wake Dynamics Changes during Maturation in Rats
Source: PLoS One. 2015 Apr 20;10(4):e0125509. doi: 10.1371/journal.pone.0125509 (PMC4404317; doi:10.1371/journal.pone.0125509)

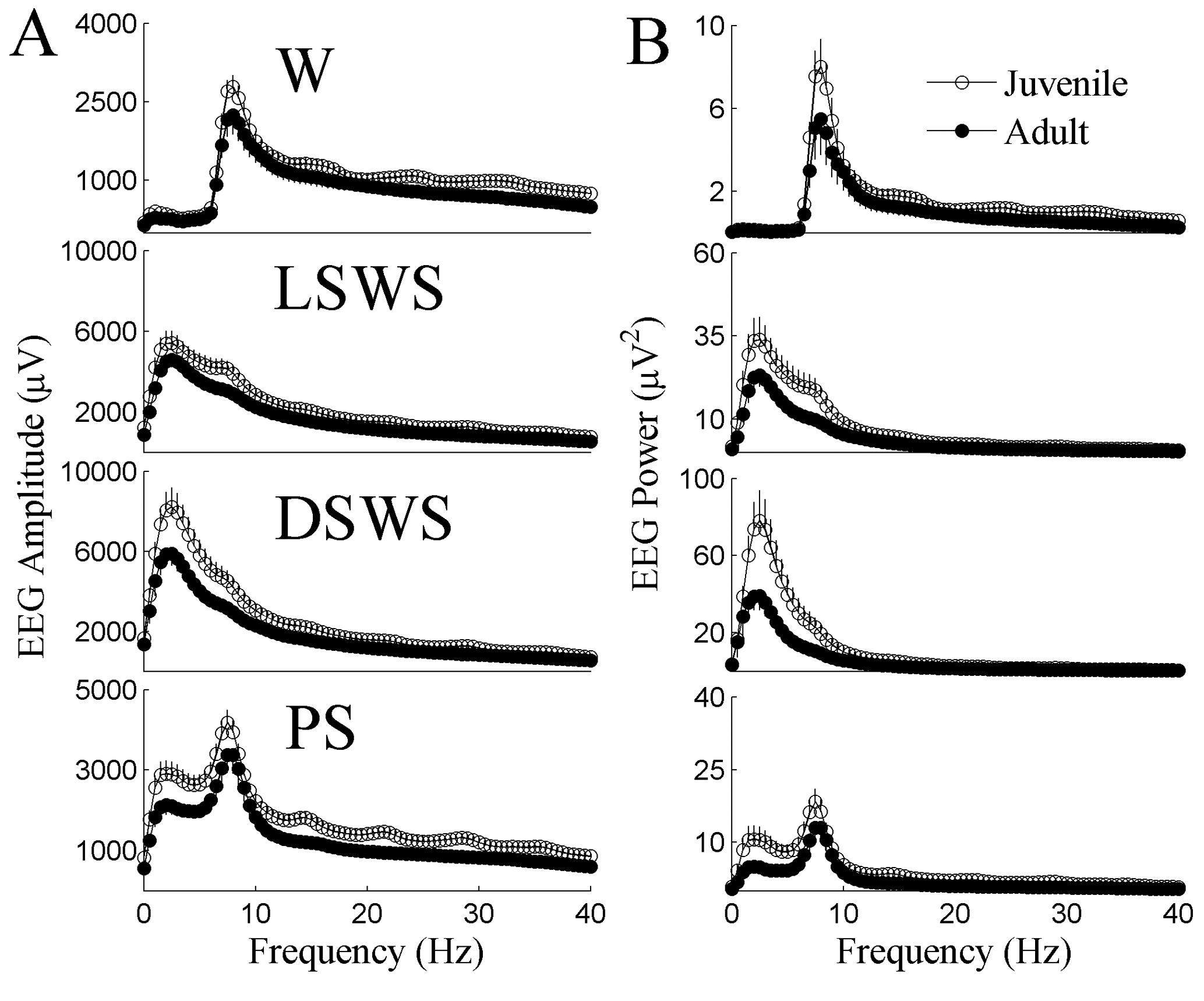

Supplement: S1 Fig — Values were calculated in wake (W), light slow wave sleep (LSWS), deep slow wave sleep (DSWS), and paradoxical sleep (PS) during 24 hrs in juvenile (open symbols) and adult (closed symbols) rats. Each point represents mean values of 0.5 Hz bins in a 24-hr recording period. There are statistically significant differences between the groups in both amplitude and power density at all frequencies in all vigilances states, p < 0.0001 (two-way ANOVA). Values are mean ± SEM. (TIF) [file pone.0125509.s001.tif]

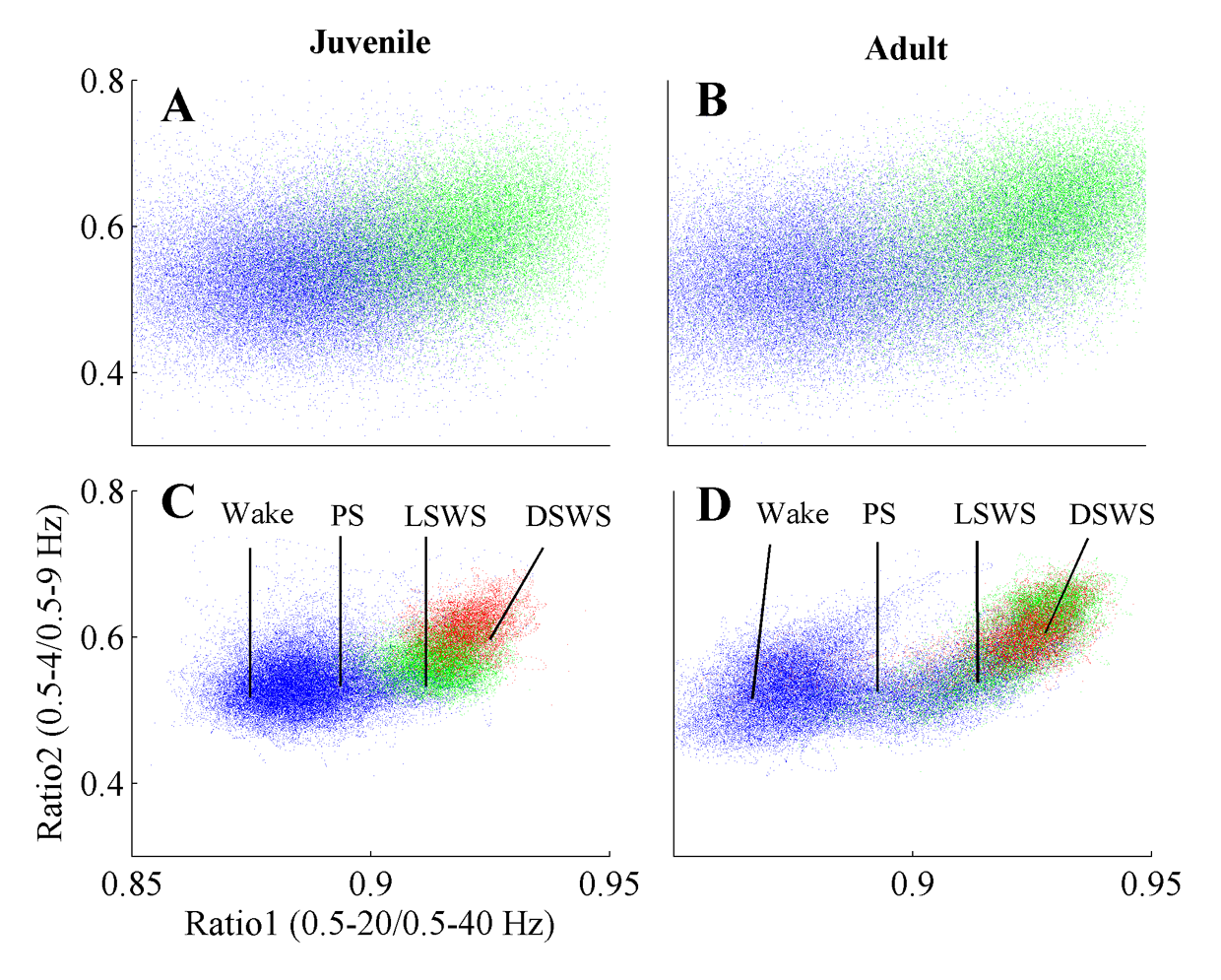

Supplement: S2 Fig — (A) Juvenile and (B) adult rat; spectral ratios of EEG activity define a 2D state space with distinct clusters. Each plot shows 24 hours of EEG activity, and each point represents 1 second of EEG activity. (C) and (D) show 2D state space after application of a Hanning window (20 seconds) of one juvenile and one adult animal, respectively. Blue—wake; Black—paradoxical sleep (PS). Green—light slow wave sleep (LSWS); Red—deep SWS (DSWS). (TIF) [file pone.0125509.s002.tif]
